# Draft Genome Sequences of 11 *Staphylococcus epidermidis* Strains Isolated from Wild Mouse Species

**DOI:** 10.1128/genomeA.01148-13

**Published:** 2014-01-16

**Authors:** Jun Wang, Sven Kuenzel, John F. Baines

**Affiliations:** Max Planck Institute for Evolutionary Biology, Plön, Germanya; Institute for Experimental Medicine, Christian-Albrechts-University (CAU) of Kiel, Kiel, Germanyb

## Abstract

We report here the draft genome sequences of 11 strains of *Staphylococcus epidermidis*, a common bacterium inhabiting the skin of humans and other animals. These isolates, obtained from five mouse species, provide valuable information on the native *Staphylococcus* spp. of this important model organism and form a basis for studying host-bacterial interactions in their natural environment.

## GENOME ANNOUNCEMENT

The genus *Staphylococcus* contains important members of the human skin microbiome (1). The major members are commensal under normal circumstances but can also be pathogenic. *Staphylococcus aureus* is so far the main species of interest, as it is a major source of nosocomial infections and can afflict numerous organs (2). Other examples include *Staphylococcus haemolyticus* (causing infective endocarditis [3]) and *Staphylococcus saprophyticus* (causing urinary tract infections [4]). *Staphylococcus epidermidis*, on the other hand, is of critical importance, as it is the most common source of medical device-associated infections (5), but at the same time, it is capable of inhibiting *S. aureus* colonization in human nasal cavities (6). Given this important clinical relevance, infection models are established in mice but are so far limited to human *S. aureus* isolates (7). Understanding the interaction and coevolutionary history between mice and their native bacterial species has attracted recent attention (8), although native *Staphylococcus* strains remain unexplored. Furthermore, we recently discovered *Staphylococcus* to contain important members of the native mouse skin microbiota influencing susceptibility to autoimmune skin blistering (9).

In order to provide insight into the native species of *Staphylococcus* inhabiting mice, we isolated *Staphylococcus* spp. from 11 wild mice representing five species and subspecies (*Mus musculus musculus, M. musculus domesticus, M. musculus castaneus, M. musculus spicilegus*, *and Apodemus uralensis*), which were captured from the wild and maintained in conventional animal facilities at the Max Planck Institute for Evolutionary Biology, Ploen, Germany. The majority of isolates belonged to *S. epidermidis*, and we subsequently selected 11 strains for genome sequencing. The sequencing libraries were prepared using the Illumina Nextera XT kit and run on the MiSeq platform with paired-end reads of 250 bp, with a minimum coverage of 31× and a maximum of 61×. The reads were assembled *de novo* using Velvet (10) with parameters optimized by VelvetOptimiser (http://www.vicbioinformatics.com/software.velvetoptimiser.shtml). The contigs were annotated by the NCBI Prokaryotic Genome Annotation Pipeline (PGAP) version 2.0 (11).

For the 11 strains, we obtained a minimum of 91 and maximum of 277 contigs, and the total number of assembled nucleotides ranged from 2,458,755 to 2,762,809 per strain. The average G+C contents ranged from 31.7% to 32.0%, which is close to those of the available reference strains (32.1% for *S. epidermidis* ATCC 12228 and 32.2% for *S. epidermidis* RP62A). A range of 2,259 to 2,541 proteins were predicted and annotated using the NCBI PGAP, with 83.6% to 92.2% of the proteins having homologs in the *S. epidermidis* ATCC 12228 (NCBI accession no. NC_004461) (12) and/or RP62A genomes (NCBI accession no. NC_002976) (13) (BLASTp [14] with an *E* value of 1E-20 and similarity threshold of 0.8). Thirteen to 70 tRNA genes and 3 to 24 rRNA genes are predicted for each strain. Further analyses of the genomic content may reveal important aspects of the interaction and coevolution of *S. epidermidis* and mouse hosts.

### Nucleotide sequence accession numbers.

The draft genome sequences are deposited in GenBank under accession no. ATCU00000000, ATCV00000000, ATCW00000000, ATCX00000000, ATCY00000000, ATDA00000000, ATDC00000000, ATDE00000000, ATDF00000000, ATDG00000000, and ATDH00000000. The second versions are described in this paper: ATCU02000000, ATCV02000000, ATCW02000000, ATCX02000000, ATCY02000000, ATDA02000000, ATDC02000000, ATDE02000000, ATDF02000000, ATDG02000000, and ATDH02000000.
